# The quick and the dead: when reaction beats intention

**DOI:** 10.1098/rspb.2009.2123

**Published:** 2010-02-03

**Authors:** Andrew E. Welchman, James Stanley, Malte R. Schomers, R. Chris Miall, Heinrich H. Bülthoff

**Affiliations:** 1 School of Psychology, University of Birmingham, Birmingham B15 2TT, UK; 2 School of Medicine, University of Otago, Dunedin, New Zealand; 3 Pembroke College, University of Cambridge, Cambridge CB2 1QA, UK; 4 Max Planck Institute for Biological Cybernetics, Tübingen, Germany; 5 Department of Brain and Cognitive Engineering, Korea University, Korea

**Keywords:** movement control, action observation, interpersonal competition

## Abstract

Everyday behaviour involves a trade-off between planned actions and reaction to environmental events. Evidence from neurophysiology, neurology and functional brain imaging suggests different neural bases for the control of different movement types. Here we develop a behavioural paradigm to test movement dynamics for intentional versus reaction movements and provide evidence for a ‘reactive advantage’ in movement execution, whereby the same action is executed faster in reaction to an opponent. We placed pairs of participants in competition with each other to make a series of button presses. Within-subject analysis of movement times revealed a 10 per cent benefit for reactive actions. This was maintained when opponents performed dissimilar actions, and when participants competed against a computer, suggesting that the effect is not related to facilitation produced by action observation. Rather, faster ballistic movements may be a general property of reactive motor control, potentially providing a useful means of promoting survival.

## 1. Introduction

The mythology of the American West is shaped by liquor and Hollywood (Brown 1995). Inspired at least by the latter, the Nobel laureate Niels Bohr considered why, during a gunfight, the man who drew first was the one to get shot. He suggested that the intentional act of drawing and shooting is slower to execute than the reactive action in response (Cline 1987), an idea grounded in the everyday trade-off between stimulus-driven behaviour and intentional, planned actions.

This distinction between different classes of action is not merely semantic: evidence for differential neural bases for intentional, as opposed to reactive, movements is provided by neurophysiology (Kurata & Tanji 1985; Romo & Schultz 1987; Mushiake *et al*. 1991; Maimon & Assad 2006), neurology (Laplane *et al*. 1977; Halsband *et al*. 1993; Cunnington *et al*. 1995; Sumner *et al*. 2007) and functional brain imaging (Deiber *et al*. 1999; Jenkins *et al*. 2000; Cunnington *et al*. 2002). Further, behavioural evidence points to a distinction between different types of movement (Waszak *et al*. 2005), and switching between these two modes of operation can result in a cost (Obhi & Haggard 2004). However, here we test whether there are benefits associated with reactive movements, consistent with Bohr's intuition and the gunslingers legend.

**Figure 1.**
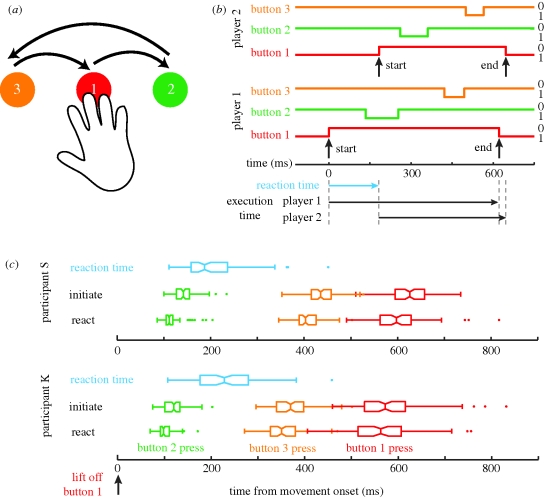
(*a*) An illustration of the button press sequence. Button 1 was referred to as the ‘home key’ and participants initiated a trial by keeping this button depressed with their right hand. They then moved to the right to hit button 2, then all the way to the left to hit button 3, before returning to button 1. Buttons were separated laterally by 35 cm (experiment 1) or 15 cm (experiments 2, 3), meaning that arm movement was necessary. (*b*) An illustration of a single trial competition between two participants. Players had their own set of three buttons. The movement sequence starts by one player lifting their hand off button 1, and ends by pressing button 1 again having meanwhile pressed buttons 2 and 3. In this trial player 1 was the initiator and player 2 the reactor: player 1's button 1 is lifted up before player 2's. Player 1 completes the movement sequence first but player 2 executes the movement faster. Note that this difference in execution times could be spurious: player 2 might simply make faster movements. Thus, we compared movement times from the same participant—contrasting trials when they were the initiator with those in which they were the reactor. (*c*) Distributions of button press times for two representative participants. Boxplots depict the median, interquartile range and the extreme values; outliers are shown as single points; notches show 95% CI for the median. The blue boxplots show the distribution of reaction times on ‘reactive’ trials. The green, orange and red boxplots show the times at which participants depressed buttons 2, 3 and 1, respectively. Separate series are used for reactive and initiative trials. All times are relative to releasing button 1. As expected for time data, distributions are positively skewed (Ratcliff 1993). The increasingly broad distributions for buttons 3 and 1 are expected as time is relative to button 1 being released, so variation is compounded at each subsequent stage.

To effect ‘laboratory gunfights’, we devised a relatively simple task of button pressing that required a stereotyped, multi-segment movement. In particular, naive participants made a speeded sequence of three button presses that required a lateral movement of their hands (figure 1*a*). The movement direction and sequence of button presses was the same on every trial. Having become familiar with this task, participants were paired with an opponent and placed in competition (figure 1*b*). Opponents faced each other with their own set of buttons before them and held down the central button (button 1, ‘the home key’) to start a trial. They were instructed that by executing the movement and returning to their home key before their opponent, they would score points from their adversary.

To distinguish ‘initiated’ from ‘reactive’ movements, we had to ensure that trials had no overt ‘go’ signal; otherwise, all subsequent movements could putatively be ‘reactive’. Therefore, participants were forced to wait a variable, non-signalled delay before initiating the movement. If participants released the home key too early, a tone sounded and the trial was aborted. The covert and random nature of the start delay, and competition between opponents, meant that each individual produced some trials on which they initiated the movement sequence (‘initiated’ movements) and other trials on which they reacted to their opponent (‘reactive’ movements). Data analysis considered within-subject differences in movement execution times. That is, we compared the response of each individual under initiative and reactive movement conditions, rather than considering the relative performance of different participants and/or the outcome of interpersonal competitions. We report results from three experiments. The first establishes the effect; the second tests whether faster responses result from observing the movement of the opponent, and the third tests the importance of a social context.

## 2. Material and methods

### (a) Participants

Participants were able-bodied, naive individuals (aged 18–39) recruited from subject pools in Tübingen and Birmingham. Ten participants (four males, six females) were used for experiment 1, 10 participants for experiment 2 (six males, four females), and 14 for experiment 3 (one male, 13 females). Further, groups of eight (six males, two females) and 12 (seven males, five females) participants were used in additional experiments. All gave written informed consent and local ethics committees approved the experiments.

### (b) Equipment

The equipment consisted of two sets of three buttons interfaced with a PC through a data acquisition card (experiment 1) or the parallel port (experiments 2, 3). Each participant had a set of buttons attached to the table on which they sat. The buttons were capacitor-based switches encased in a rigid plastic of 4.5 cm diameter (Captronic Electronic GmbH), i.e. there were no moving parts and the buttons did not physically change when touched. Custom-built electronics converted the button output to a standard 5 V pulse. Button presses were detected reliably (*σ* < 1.2 ms) with negligible lag. The centre-to-centre spacing of the buttons was 35 cm (experiment 1) or 15 cm (experiments 2, 3). Participants sat at either end of a 140 cm long table. Only for experiment 3, a 20-inch LCD located 120 cm from the participant was used for the visual display of symbolic button presses. In particular, a row of three squares on a black background was presented (one for each button, spatially arranged to correspond to a real opponent). The colour of these squares changed from red to white to depict the periods during which the opponent was pressing the button. The behaviour of the opponent was thus marked in an abstract manner that reflected only the state of the buttons (i.e. there was no movement and no schematic illustration of the opponent).

### (c) Procedure

Participants initially took part in a training session to familiarize themselves with the task. They were instructed to start a trial by resting their right-hand on the central button (the ‘home key’), then move to hit the button on their right, then on their left, and then return to the central button and keep it depressed (figure 1*a*). Participants were informed that a variable delay was imposed on each trial (no details given), so they had to wait for some time before starting their movement. The random start delay was drawn from a normal distribution (*μ* = 2500 ms; *σ* = 500 ms). Moving too early caused a warning tone, indicating an early movement error. If participants missed a button, or hit buttons out of sequence, a different tone sounded. On the basis of either error type, the trial was aborted and then repeated.

In competitive situations, participants sat facing another human player, or a display depicting their opponent's button presses. Testing sessions lasted approximately 1 h, yielding around 170 data points per condition per participant for experiment 1, 100 for experiment 2 and 120 for experiment 3. The relative number of initiated and reactive movements within this total varied between participants (i.e. as a dynamic competition, this depended on the behaviour of individuals). In most situations, participants completed a side-to-side movement. However, experiment 2 also considered front–back movements. Here, the board on which the buttons were mounted was rotated by 90° and re-attached to the table, aligned to the participant's midline.

### (d) Data analysis

Response times and movement execution times were calculated using signals from the capacitive buttons. The ‘reaction time’ was defined as the time difference between the first participant's centre button switching to an off (low) state, and their opponent's centre button switching to an off state (figure 1*b*). The ‘execution time’ for the first movement phase was defined as the time between the centre button being in a low (off) state and the right button being in a high (on) state. Subsequent movement phases were similarly calculated, with the total execution time defined as the time between the centre button being low (movement start) and high (movement end). Our use of capacitive buttons meant that downward force was not required—rather light touch was sufficient to keep buttons in a high (on) state, thus movement onset was defined as the moment at which the hand moved away from the buttons.

Data analysis considered the distribution of movement execution times produced when participants moved before (initiated movements) or after (reactive movements) their opponents (figure 1*c*). Following Luce (1986), we quantified these distributions using the harmonic mean to provide a robust statistic suitable for non-Gaussian data (in fact, using the arithmetic mean or the median made little difference). As participants could independently elect to initiate a given trial at very similar times, some ‘reactive’ movements might have in fact been ‘initiated’. We therefore discarded trials on which a participant's reaction time was below 100 ms (6.8% trials), reasoning that anything faster would be unlikely to result from a reaction (note that this 100 ms exclusion criteria corresponds to the time difference between the buttons being lifted, rather than any aspect of the opponent's behaviour that might signal their intention to move). (We ensured that this 100 ms exclusion criterion did not bias our findings by analysing our data without constraining the reaction time, and the results were unchanged.) We used further criteria to deal with outliers in the movement times (Ratcliff 1993). One possibility was that a ‘reactor’ would miss their opponent's movement, responding with a considerable delay and thus in a non-competitive manner. To avoid this possibility, data were excluded if the reaction time exceeded 500 ms (3.5% of trials). Finally, on some trials a participant would complete the sequence by correcting for a missed button, producing a long, uncompetitive execution time. Therefore, trials on which the execution time exceeded 1000 ms (experiment 1) or 800 ms (experiments 2, 3) were excluded (0.5% of trials). (Note that the larger movement amplitude required in experiment 1 produced longer execution times.) While error rates were generally low, some participants produced an unacceptably large number of errors, making their data unreliable. We excluded one subject from experiment 2, and four subjects from experiment 3 because of a high proportion of slow reaction and slow execution errors (defined as more than 25% errors in two or more conditions).

## 3. Results

### (a) Experiment 1

**Figure 2.**
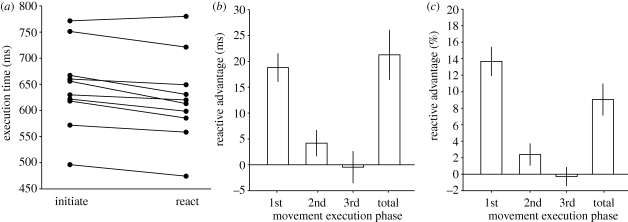
(*a*) Average movement execution times for each individual participant. Data show the harmonic mean execution time. Points connected by lines indicate the data from a single individual. Our data analysis considered the difference between these matched-pair responses. (*b*) The ‘reactive advantage’ (=initiated movement execution time−reactive movement execution time) for the three component phases of the movement sequence (1st: lift up from button 1, press down button 2; 2nd: lift up button 2, press down button 3; 3rd: lift up button 3, press down button 1), and for the total execution time (lifting up button 1 to pressing it down again having pressed button 2 and then 3). Data illustrate the between-subjects mean response. Error bars show s.e.m. (*c*) The reactive advantage expressed as a percentage change in the mean execution time. Data illustrate the between-subjects mean response with error bars showing s.e.m.

To investigate whether there was an advantage for reactive movements, we considered within-subject differences in movement execution times for trials on which participants initiated the movement sequence compared with trials on which they reacted following the movement of their opponent (figure 1*c*). We found that execution times were quicker by an average of 21 ms when participants reacted to their opponent's movement (figure 2*a*; *t*_9_ = 4.406, *p* = 0.002), an improvement of around 9 per cent. This ‘reactive advantage’ was most pronounced for the first movement of the three-button press sequence (figure 2*b*,*c*), quickening responses by around 14 per cent of the mean movement execution time. Moreover, the advantage was maximal when participants moved approximately 200 ms after the opponent (electronic supplementary material). However, as the reactive advantage in movement execution (mean = 21 ms) was less than the participant's reaction time to the movement of their opponent (mean = 207 ms), reactors rarely beat initiators (e.g. compare the difference between the red boxplots for a participant with the extent of their reaction time (blue boxplots) from figure 1*c*).

The proportion of failures to hit one of the buttons in the sequence increased for reactive movements, suggesting that increased speed is associated with reduced accuracy (Woodworth 1899). This in and of itself does not constitute a trivial explanation for our findings, as participants did not know *a priori* whether a reactive or intentional movement would be made. Had the roles of initiator and reactor been predetermined at the start of a trial, data interpretation would be complicated. In particular, if participants knew that they would react on a given trial, they could consciously elect to maximize their chances of winning the duel by producing faster and less accurate movements to compensate for the time cost of reacting after their opponent. However, the dynamic nature of the competition meant that this was not possible: both participants prepared to initiate the movement sequence. Thus, like a quickening of execution times, a change in error rates suggests a dynamic computation of movement influenced by the type of movement being produced. High-speed, low-accuracy movements may constitute a characteristic of the neural systems responsible for controlling reactive movements.

A potentially trivial difference between movements executed under reactive and intentional conditions is that, when making a reactive movement, participants had been waiting longer to move. Under some circumstances preparation time and movement speed are related (Rosenbaum *et al*. 1987; Bullock & Grossberg 1988), potentially suggesting a lurking variable between our two movement classes. Our use of the covert random start delay made this suggestion rather unlikely (the time from the trial starting to the initiating participant's movement had a between-trial standard deviation of 775 ms in contrast to typical reaction times of 200 ms). However, we tested this idea by performing regressions of movement execution time on the initiation time on a per subject basis, finding no evidence for the relationship between preparation and execution times under our experimental paradigm.

An additional concern might relate to the warning tone that indicated participants had moved before the end of the covert delay period. In particular, the tone might effectively act as a ‘penalty’ that could change the movement strategy so that participants were cautious, and thus slower, when initiating the movement sequence. To test this idea, we ran a control experiment on eight participants in which the no intertrial delay was imposed. We found clear evidence for a reactive advantage (*t*_7_ = 8.426, *p* < 0.001) when there was no warning tone, ruling out this concern and suggesting that any penalizing effect of the warning tone was not responsible for faster reactive movements. These data were also useful in allowing us to test for evidence of a speed-accuracy trade-off under our paradigm (we could not do this for the main experimental data as button press times were not recorded for error trials). In particular, we considered the duration of the first movement in the sequence of trials in which an error was subsequently made (e.g. the participant missed button 3 or 1 during subsequent movements). We compared the duration of these movements with those measured on successful (non-error) trials, to test whether errors were associated with faster movements. We found no evidence for a difference between error and non-error movement times for either reactive (*p* = 0.27) or intentional (*p* = 0.80) movement sequences.

Finally, our task requiring a movement sequence of three distinct segments might be regarded as overly complex, with the necessity of reversing the direction of travel leading to uncertainty in hand position after the initial movement has been made. In an additional control experiment we asked participants to make a simple, single segment movement (from button 3 to button 2) under competition. Consistent with our previous findings, we observed clear evidence for a reactive advantage (*t*_7_ = 3.852, *p* = 0.006) for this simple ballistic movement.

### (b) Experiment 2

To gain further insight into the reactive advantage, we asked whether the effect might accrue from having the opponent's movement as a model for one's own actions. In particular, ventral premotor cortex is known to be activated similarly when a participant performs an action or observes the same movement performed by another actor (Gallese *et al*. 1996; Iacoboni *et al*. 2005), potentially priming movement production circuits and facilitating the participant's own actions. Behaviourally, movement production can be influenced by viewing an action that is either incongruent with one's own (Brass *et al*. 2000) or a transformed version of what one has to perform (Craighero *et al*. 2002).

**Figure 3.**
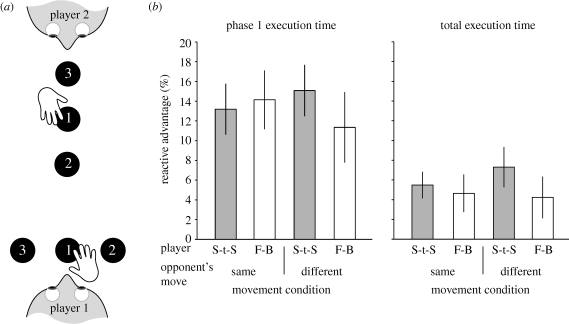
(*a*) Aerial view illustrating the set-up used in experiment 2. The row of buttons could be placed horizontally (illustrated for player 1 who would make a side-to-side movement, white bars) or vertically (illustrated for player 2 who would make a front–back movement, grey bars). The two players could make the same type of movement (as in experiment 1) or a different type of movement (as illustrated in the cartoon). (*b*) The reactive advantage (expressed as a percentage) for phase 1 and total execution times in experiment 2. Data illustrate the between-subjects mean response with error bars showing s.e.m. Data from the four different movement conditions are shown.

To investigate the possibility that the opponent's movement facilitates movement production, we tested whether the direction in which participants moved influenced the advantage for reactive movements. In particular, participants performed the three-button press sequence when the buttons were configured for side-to-side or front–back movements (figure 3*a*). Thus, a player could see their opponent making a comparable movement that could act as a model for their own movement (e.g. both players make a front–back movement), or movement orthogonal to their own action that would be of less use in priming action preparation (e.g. one player moves front–back while the other moves side-to-side). Consistent with our previous experiments, we observed a significant decrease in execution times for reactive movements (*F*_1,8_ = 11.484, *p* = 0.01). However, there was no significant effect of viewing a different movement from one's own (*F*_1,8_ = 3.273, *p* = 0.108) or any significant interactions (figure 3*b*). Thus, the reactive advantage does not appear to be modulated by viewing the opponent making similar or dissimilar movements.

### (c) Experiment 3

In our final experiment, we tested whether the social context within which the participants found themselves might be responsible for their facilitated reactive movements. Previous work suggests differential performance when humans believe they are interacting with another human compared with a non-human agent such as a computer, based on the notion that the mirror neuron system acts to determine the intentions of others (Kilner *et al*. 2003; Stanley *et al*. 2007; Gowen *et al*. 2008). To examine the role that might be played by cortical systems responsible for encoding the intentions of others, we contrasted performance when participants competed against another human (‘Person’ condition) or a computer on whose display movements were presented symbolically. In computer opponent conditions, participants were informed either (i) that they were competing against a computer (‘computer’ condition), or (ii) that they were competing against another human located in a different testing room, interfaced through the computer (‘virtual’ condition). In actuality, the distribution of movement onset and movement execution times produced by the computer was determined from data previously recorded from the participant, meaning that they were playing against a historical version of themselves, and thus involved in a demanding competition. Debriefing participants at the end of the session revealed this manipulation to have been successful, with only one participant expressing doubts about the authenticity of their computer-interfaced human opponent.

**Figure 4.**
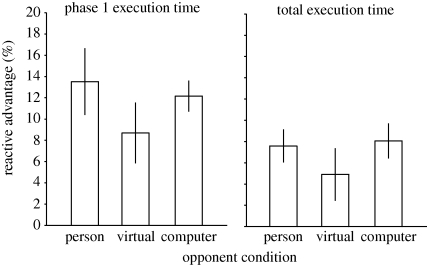
The reactive advantage (expressed as a percentage) for phase 1 and total execution times in experiment 3. Data illustrate the between-subjects mean response with error bars showing s.e.m. Data from the three different movement conditions are shown: human opponent, virtual opponent (in which the player faces a computer but is told that this interfaces with another human) and computer opponent (participants are told that they are playing with a computer).

Consistent with the previous experiments, faster movements were observed under reactive conditions (*F*_1,9_ = 26.689, *p* = 0.001). However, the type of opponent faced by participants (human, computer, virtual human) neither had significant influence on execution times (*F*_2,18_ = 2.967, *p* = 0.077) nor was the interaction between the reactive advantage and the type of opponent significant (*F*_2,18_ = 1.650, *p* = 0.220). The statistical analysis on the type of opponent might suggest a marginal effect. Nevertheless, inspecting the data (figure 4) does not suggest a pattern of results consistent with the hypothesis that the anthropomorphic nature of the opponent modulates the effect. In particular, if this hypothesis underlies the effect, we would expect the reactive advantage to be lowest for conditions in which the player believed they were competing with a computer. Rather, the mean reactive advantage is lowest when participants believed they were competing against another human interfaced through the computer.

Despite this null result, the findings from this experiment are useful in addressing concerns that might arise from the paradigm we have developed. In particular, under conditions in which participants compete against a computer, there is no visual motion and no auditory cues from the opponent, nor any cues of movement preparation. A previous report suggests that movements are faster when participants are able to see motion (Smeets & Brenner 1995), suggesting an alternative interpretation for a benefit of viewing the opponent. However, this cannot explain our findings as the reactive advantage persists when there is no motion in the display (just three simple illuminated squares). Moreover, auditory cues from the human opponent hitting their buttons could increase arousal, or provide a movement-timing signal. However, such cues are not available when competing against a computer, as there was no physical movement or button pressing. Nevertheless, our findings were unchanged. Finally, in the context of bimanual reaching, it is known that participants slow down the easier of two movements when reaching for two different targets, such that both movements end simultaneously (Kelso *et al*. 1979). Similar effects of movement coordination have been reported for social (two-person) movements (Georgiou *et al*. 2007). It could be argued that intentional movements are deliberately slowed to ensure synchronous termination with an opponent's reactive movement. The competition between participants and the considerable delay imposed by reaction make this suggestion unlikely. Moreover, the sparse display in computer-opponent conditions effectively rules out this possibility.

## 4. General discussion

Here we consider the production of the same movement sequence under conditions in which participants initiate the movement or react to an opponent. We demonstrate that reactive movements are associated with faster execution times, and that this quickening of movement does not appear to relate to having another human as a model for one's own action. We suggest different cortical processing routes for the control reactive versus intentional movements, and argue that faster movement dynamics may constitute a basic property of reactive movement production.

The suggestion of a distinction between reactive versus intentional movements is consistent with a range of previous studies that report changes in the balance of the involvement of a number of cortical and subcortical areas during the production of different classes of action (Laplane *et al*. 1977; Kurata & Tanji 1985; Romo & Schultz 1987; Mushiake *et al*. 1991; Halsband *et al*. 1993; Cunnington *et al*. 1995, 2002; Deiber *et al*. 1999; Jenkins *et al*. 2000; Maimon & Assad 2006; Sumner *et al*. 2007). Previous behavioural work also supports this distinction. For instance, countermanding the production of an intended movement to react to an external trigger can have a cost (Obhi & Haggard 2004), suggesting a delay imposed by switching between different modes of movement triggering (Obhi *et al*. 2009*b*). Under our paradigm, participants could be provoked to move sooner than they intended by seeing their opponent's actions. Based on Obhi and colleagues' findings, the initiation of such reactive movements may be slower than the initiation of internally generated movements. (This suggestion is, of course, untestable as we have no access to the timing of participant's movement triggering decisions.) Here, we assess a different aspect of movement production, demonstrating that reactive movements can be advantageous in producing faster execution times (albeit with increased error rates).

It is interesting to speculate about the neural circuits that might be involved in the production of the movements we have studied. One candidate region of importance is the pre-supplementary motor area (SMA) region of the medial frontal cortex that is implicated in the control of intentional actions. Moreover, it is thought to play a key role in switching between different tasks (Rushworth *et al*. 2002) and selecting an intentional action over a reactive one (Isoda & Hikosaka 2007). It is possible that the pre-SMA functions to remove the inhibition of potential actions—a function carried out by the SMA (Sumner *et al*. 2007)—thereby giving rise to the production of the intentional movement sequence. The production of reactive movements may involve an alternative route that disinhibits the planned movement sequence via the parietal cortex (Cunnington *et al*. 2006). Our observation that the reactive advantage is focused on the initial, ballistic phase of movement is suggestive of an effect limited to movement onset rather than being general to the production of a sequence of arm movements. This suggests different types of disinhibition for reactive and intentional movements. In particular, disinhibition designed to prevent early movement (Sinclair & Hammond 2009; see Obhi *et al*. 2009*a* for an excellent discussion) may be faster via the parietal route, resulting in increased acceleration and reduced movement execution times.

Faster movement speed for reactive movements has a parallel to known deficits in Parkinson's disease. In particular, Parkinson's patients are especially compromised in speed when making intentional, rather than reactive, reaching arm movements (Majsak *et al*. 1998). Differences between reactive and intentional movement systems may thus become more apparent in Parkinson's as the basal ganglia makes a greater contribution to intentional actions (Roland *et al*. 1982; Jones 1987). Testing Parkinson's patients with our paradigm would be of interest as concerns about high-level speed-accuracy decisions, or strategies for different experimental (i.e. self-paced versus stimulus-driven) conditions could be ruled out.

We interpret our results as reflecting the operation of different processing routes for intentional versus reactive movements; however, might the results rather reflect a deliberate strategy by participants, and thus not imply different neural architectures? In particular, perhaps participants deliberately change their movements according to whether a reactive or an intentional movement is required, optimizing their actions by speeding up on reactive trials when there is less chance of ‘winning’ the duel, and slowing down on intentional trials to minimize energetic cost. This would be possible had participants known ahead of time whether an intentional or a reactive movement was required. However, under our paradigm, the dynamic nature of the competition meant that participants have little opportunity to change their movements deliberately, as on any given trial they might be the initiator or the reactor, and reaction times were low (*ca* 200 ms).

It could also be argued that individuals learn about their opponent's behaviour across trials, thereby developing a strategy based on the probability of making an intentional or reactive movement on a particular trial. We believe this is unlikely as there was no feedback at the end of each trial and participants were thus very frequently unaware of who had won the duel (detecting the small temporal offsets between one's own actions and that of the opponent in the context of a rapid competition was not easy). Moreover, under some circumstances (experiment 3), participants were effectively playing against themselves, making it difficult to argue that participants exploited differences between their own behaviour and that of their opponent to maximize their chance of winning the duel. However, we tested whether there was a systematic relationship between the probability of being a ‘reactor’ and the reactive advantage. (Data were pooled across experiments to maximize statistical power, and the reactive advantage was expressed as a percentage to minimize the influence of between-subject differences in movement times.) We found no evidence of a relationship between the probability of reacting and the increased speed of reactive movements (*r* = 0.11, *F*_1,39_ < 1, *p* = 0.485).

As a general survival strategy, the evolution of a movement system capable of producing quick (and possibly dirtier) movements that support faster responses to the environment seems reasonable. However, within the context of a gunfight, a strategy based purely on reaction seems unlikely to increase evolutionary fitness as the advantage produced by reacting is far outweighed by the time taken to react to the opponent. Anecdotal reports suggest that Bohr tested his original idea with colleague George Gamow using toy pistols, with the ‘reactive’ Bohr apparently winning every duel (Cline 1987). Our data make it unlikely that these victories can be ascribed to the benefits associated with reaction. Rather, they suggest that Bohr was a crack shot, in addition to being a brilliant physicist.
